# Phlebotomine sand flies (Diptera: Psychodidae) transmitting visceral leishmaniasis and their geographical distribution in China: a review

**DOI:** 10.1186/s40249-016-0107-z

**Published:** 2016-02-23

**Authors:** Li-Ren Guan, Zheng-Bin Zhou, Chang-Fa Jin, Qing Fu, Jun-Jie Chai

**Affiliations:** National Institute of Parasitic Diseases, Chinese Center for Disease Control and Prevention/Key Laboratory of Parasite and Vector Biology, Ministry Of Health/WHO Collaborating Center for Tropical Diseases, Shanghai, 200025 China; Center for Disease Control and Prevention of Xinjiang Uygur Autonomous Region, Urumqi, 830002 China

**Keywords:** *Phlebotomus*, Sand fly, Geographical distribution, Vector, Visceral leishmaniasis, *Leishmania donovani*, *Leishmania infantum*, *Ph. chinensis*, *Ph. longiductus*, *Ph. wui*, *Ph. alexandri*, *Ph. mongolensis*

## Abstract

After the existence of phlebotomine sand flies was first reported in China in 1910, the distribution of different species and their role in the transmission of visceral leishmaniasis (VL) have been extensively studied. Up until 2008, four species have been verified as vectors of VL, namely, *Phlebotomus chinensis* (*Ph. sichuanensis*), *Ph. longiductus* (*Ph. chinensis longiductus*), *Ph. wui* (*Ph. major wui*), and *Ph. alexandri.*

The sand fly species vary greatly depending on the natural environments in the different geographic areas where they are endemic. *Ph. chinensis* is euryecious and adaptable to different ecologies, and is thus distributed widely in the plain, mountainous, and Loess Plateau regions north of the Yangtze River. *Ph. longiductus* is mainly distributed in ancient oasis areas south of Mt. Tianshan in the Xinjiang Uygur autonomous region. *Ph. wui* is the predominant species in deserts with *Populus diversifolia* and *Tamarix* vegetation in Xinjiang and the western part of the Inner Mongolia autonomous region. Finally, *Ph. alexandri* is steroecious and found only in stony desert areas, such as at the foot of the mountains in Xinjiang and the western Hexi Corridor, in Gansu province. This review summarized the relationship between the geographic distribution pattern of the four sand fly species and their geographical landscape in order to foster research on disease distribution and sand fly control planning. Furthermore, some problems that remained to be solved about vectors of VL in China were discussed.

**Electronic supplementary material:**

The online version of this article (doi:10.1186/s40249-016-0107-z) contains supplementary material, which is available to authorized users.

## Multilingual abstracts

Please see Additional file 1 for translation of the abstract into the six official working languages of the United Nations.

## Introduction

After the existence of phlebotomine sand flies was first reported in China in 1910 [1], the Western scholars Young and Hertig conducted an experiment to understand the sand fly species *Ph. chinensis* and *Ph. mongolensis* and their transmission of *Leishmania* in Xuzhou, Jiangsu province in 1925. As a result, they pioneered research on vectors of visceral leishmaniasis (VL) in China [2]. Subsequently, scientists in the country have made substantial contributions to determining which sand fly species are vectors of VL. From 1936 to 1941, during World War II, scientists were for the first time able to determine, through field investigation and experimental research, that *Ph. chinensis* was a vector of VL in the plain areas of north China. Since 1964, the successors of these scientists have been able to prove that *Ph. longiductus, Ph. wui*, and *Ph. alexandri* are all vectors of VL in Xinjiang, Inner Mongolia, and other areas in China. The geographic distribution for the four sand fly species was mapped, facilitating research on disease distribution and sand fly control planning.

## Review

### Ph. chinensis

#### Geographic distribution

**Fig. 1:**
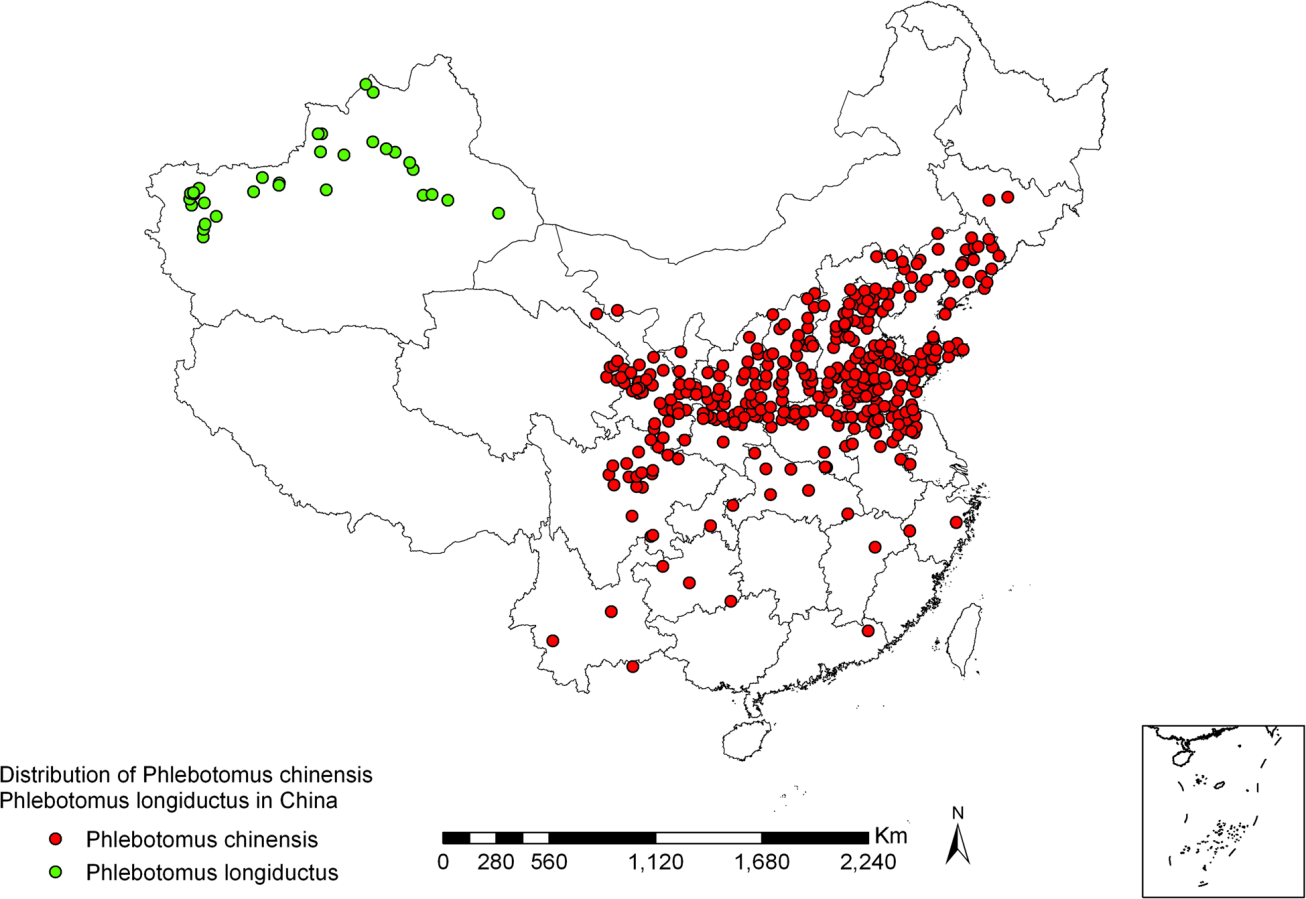
Geographic distribution of *Ph. chinensis* and *Ph. longiductus* sand flies in China

**Fig. 2:**
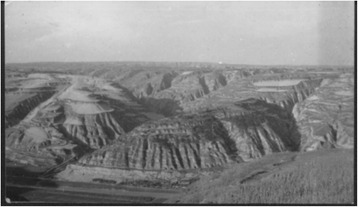
The Loess Plateau, covered with thick loess, was gradually eroded into a landscape with fragmented mountains and valleys due to long-term erosion

*Ph. chinensis* is widely distributed in China. As of 2011, it has been reported in 21 provinces, which includes 358 counties [3–5]. The species is prevalent as far north as Changchun, Jilin province (43°90′N, 125°50′E), as far south as Hekou, Yunnan province (23°40′N, 104°E), as far west as Zhangye, Gansu province (38°90′N, 100°40′E), and as far east as Jilin, Jilin province (43°80′N, 126°60′E). *Ph. chinensis* is the most predominant species in plain, mountainous, and Loess Plateau regions, in the area of 32 °– 43 ° N, 102 °–121 ° E (see Figs. 1 and 2), which is parallel to the geographical distribution of VL. According to vertical distribution surveys, *Ph. chinensis* is present at an altitude range of 10 m to 2,750 m [6], and is prevalent in the Jiangsu coastal plain and the high mountains and deep valleys of the northwestern Sichuan and southern Gansu provinces. The highest density of this species has been observed at an altitude of 1,300–1,900 m. Meanwhile, VL patients and dogs that have canine visceral leishmaniasis (CVL) were found in villages with altitudes of 1,980 m and 2,080 m, respectively [7]. *Ph. chinensis* is exophilic and widely scattered in mountainous areas and Loess Plateau [8, 9]. Due to the lack of effective measures to control wild *Ph. chinensis*, new cases of VL and CVL have frequently emerged in these terrains [10, 11].

### Vector incrimination

#### *Leishmania* obtained from VL patients was found to develop and reproduce in the alimentary tract of *Ph. chinensis* sand flies, with promastigotes migrating into the pharynx and proboscis

In 1925, Young and Hertig exposed hamsters (*Cricetulus triton*) intradermally infected with *Leishmania* isolated from VL patients to *Ph. chinensis* sand flies, and observed the development of the parasite in the sand flies. A total of 34 sand flies were fed with the hamsters’ blood. After three to eight days, the sand flies were dissected and examined; 29 (85.3 %) were found to be infected with promastigotes in the midgut and esophagus areas [2].

In the following year, Patton and Hindle performed a similar experiment in Jinan, Shandong province. After *Ph. chinensis* sand flies were fed with the blood of hamsters infected with VL or with the blood of human VL patients, the promastigotes infection rates were 77.7 % (122/157) and 4.9 % (5/102), respectively. In addition, it was observed that the promastigotes migrated to the pharynx and buccal cavity areas [12]. Since 1935, Chinese scholars had conducted this experiment in Qingjiangpu, Jiangsu province. Sun et al. fed 73 *Ph. chinensis* sand flies with the blood of VL patients. Six days later, five sand flies were found to be infected with promastigotes (6.9 %) [13].

The following year, Sun and Wu reproduced the experiment in Huai’an, Jiangsu province. The promastigote infection rates of sand flies fed on VL patients and VL hamsters were recorded as 19.3 % (26/135) and 56.3 % (36/64), respectively. *Leishmania* was seen to flourish in the stomach of the sand flies, and then migrating to the front-end of the alimentary tract [14]. Feng and Chung did a similar experiment, whereby they let *Ph. chinensis* sand flies bite four dogs with CVL in Peiping (now Beijing). Five days later, promastigotes were detected in the pharynx and proboscis areas of *Ph. chinensis* sand flies, with a high rate of infection of up to 85.8 % (103/120) [15].

Consequently, these findings indicate that *Leishmania* can develop and reproduce well in the alimentary tract of *Ph. chinensis* sand flies.

From 1958 to 1990, infection experiments were done in other areas, such as Tai’an, Lanzhou, and Jiuzhaigou, with all of them leading to the conclusion that *Ph. chinensis* is the ideal vector of *Leishmania* (see Table 1).

**Table 1:** Experimental infection of *Ph. chinensis* sand flies with blood of VL-infected patients and CVL-infected dogs (*Leishmania*) from 1958 to 1990

Year	Experimental sites (county)	Origin	No. dissected	No. infected (%)	Distribution of promastigotes in sand flies							
					Proboscis	Buccal cavity	Pharynx	Esophagus	Proventriculus	Midgut	Hindgut	Reference
1958	Tai’an, Shandong province	VL patients	177	62(35.0)	2	1	2	0	38	61	13	^a^
1960	Tai’an, Shandong province	VL patients	228	90(39.5)	1	1	4	5	71	87	13	^b^
1958	Lanzhou, Gansu province	CVL dogs	57	24(42.1)	0	0	5	0	7	19	2	^a^
1980	Lixian, Sichuan province	CVL dogs	74	45(60.8)	0	0	3	22	45	45	0	[62]
1990	Jiuzhaigou, Sichuan province	VL patients	331	229(69.2)	5	0	40	104	193	229	45	[20]

^a^Annual report, Institute of Parasitic Diseases, Chinese Academy of Medical Sciences, 1958, pp. 376–360

^b^Annual report, Institute of Parasitic Diseases, Chinese Academy of Medical Sciences, 1960, pp. 426–436

#### *Ph. chinensis* sand flies naturally infected with VL in endemic areas, and experimental infection of hamsters (*Cricetulus barabensis*)

In 1935, Sun et al., collected 421 *Ph. chinensis* sand flies from patients’ houses in Wangshiguzhuang village, Huai’an, Jiangsu province, an area where VL was prevalent. They found that seven (1.7 %) sand flies were naturally infected with promastigotes in the midgut [13]. The following year, Sun and Wu collected 537 *Ph. chinensis* sand flies from two other VL-endemic villages in the same county, and 11 (2.05 %) sand flies were found to be naturally infected with promastigotes [16]. In the same year, 11 hamsters were inoculated with promastigotes from the midgut of six sand flies by intraperitoneal injection; VL was observed in four hamsters (36.4 %) after 193 to 293 days [17].

From 1939 to 1941, Feng and Chung collected and dissected groups of 16 and 57 *Ph. chinensis* sand flies from two CVL dog nests in Beijing, and found that two (12.5 %) and 34 (59.6 %) sand flies had promastigotes, respectively. After inoculation of one hamster, *Leishmania* parasites were detected in visceral smears 10 months later [18, 19].

In 1990, naturally infected *Ph. chinensis* sand flies were found in VL-endemic areas in Jiuzhaigou county, Sichuan province [20], and promastigotes isolated using immunological methods (dot-enzyme-linked immunosorbent assay using monoclonal antibody) were identified as *L. donovani* [21]. This provided further evidence for *Ph. chinensis* sand flies being transmitting vectors.

#### Transmission of *Leishmania* from CVL-infected dogs to hamsters via *Ph. Chinensis* bites

From 1940 to 1941, Feng and Chung let *Ph. chinensis* sand flies feed on the blood of dogs infected with CVL for three days, and then unleashed 82 fed *Ph. chinensis* sand flies on caged hamsters overnight. The animals had been anaesthetized by intraperitoneal injection of urethane and abdominal skins were shaven before exposure. One of the eight hamsters (12.5 %) developed VL after being bitten by a sand fly [22]. In 1941, Ho, Chu, and Yuan were able to reproduce the same experiment. They collected *Ph. chinensis* sand flies from the field and let them feed on VL-infected hamsters and CVL-infected dogs. A week later, the sand flies were unleashed in a cage with four normal hamsters. The hamsters were dissected six months later, with *Leishmania* amastigotes found in the spleen of one hamster (25 %) [23].

Since then, Chung, Feng, and Feng developed a method that can be used to obtain a large sample of *Ph. chinensis* sand flies infected with promastigotes for a transmission experiment. During transmission season, they tied a CVL-infected dog in an empty room, leaving the doors and windows open to let sand flies in at night. The next morning, the doors and windows were shut, and the sand flies were collected and fed with raisins, pears, or apples. Some of the sand flies survived for 15 days on such diets. Seventy-two normal hamsters were then exposed to these sand flies in batches. Forty-seven hamsters were dissected after 47 to 270 days, with eight of them developing VL. The result proved beyond doubt that VL is experimentally transmissible to hamsters via the bite of *Ph. chinensis* sand flies that fed on CVL-infected dogs [24].

#### *Ph. chinensis* sand flies are widely distributed in VL-endemic areas (except for Xinjiang), and the population density of sand flies is closely related to VL endemicity

In 1937, a survey showed that a high density of *Ph. chinensis* sand flies in the total sand fly population (ranging from 84.2 % to 92.9 %) was closely correlated with VL epidemics in Huai’an, Jiangsu province. In contrast, fewer VL cases were found in villages where *Ph. chinensis* sand flies accounted only for 3–18.8 % of the total sand fly population [25].

Since the founding of the People’s Republic of China, more attention has been paid to the control of *Ph. chinensis* sand flies. As a result, data which provides further support for the correlation between VL and population density of *Ph. chinensis* sand flies, has become increasingly available*.* By the end of 1959, researchers confirmed the existence of *Ph. chinensis* sand flies in 261 of the 270 VL-endemic counties and cities in 13 provinces/autonomous regions, except for in Xinjiang. Moreover, it was found that *Ph. chinensis* was the dominant species in these counties [3].

### Ph. mongolensis

#### Geographic distribution

**Fig. 3:**
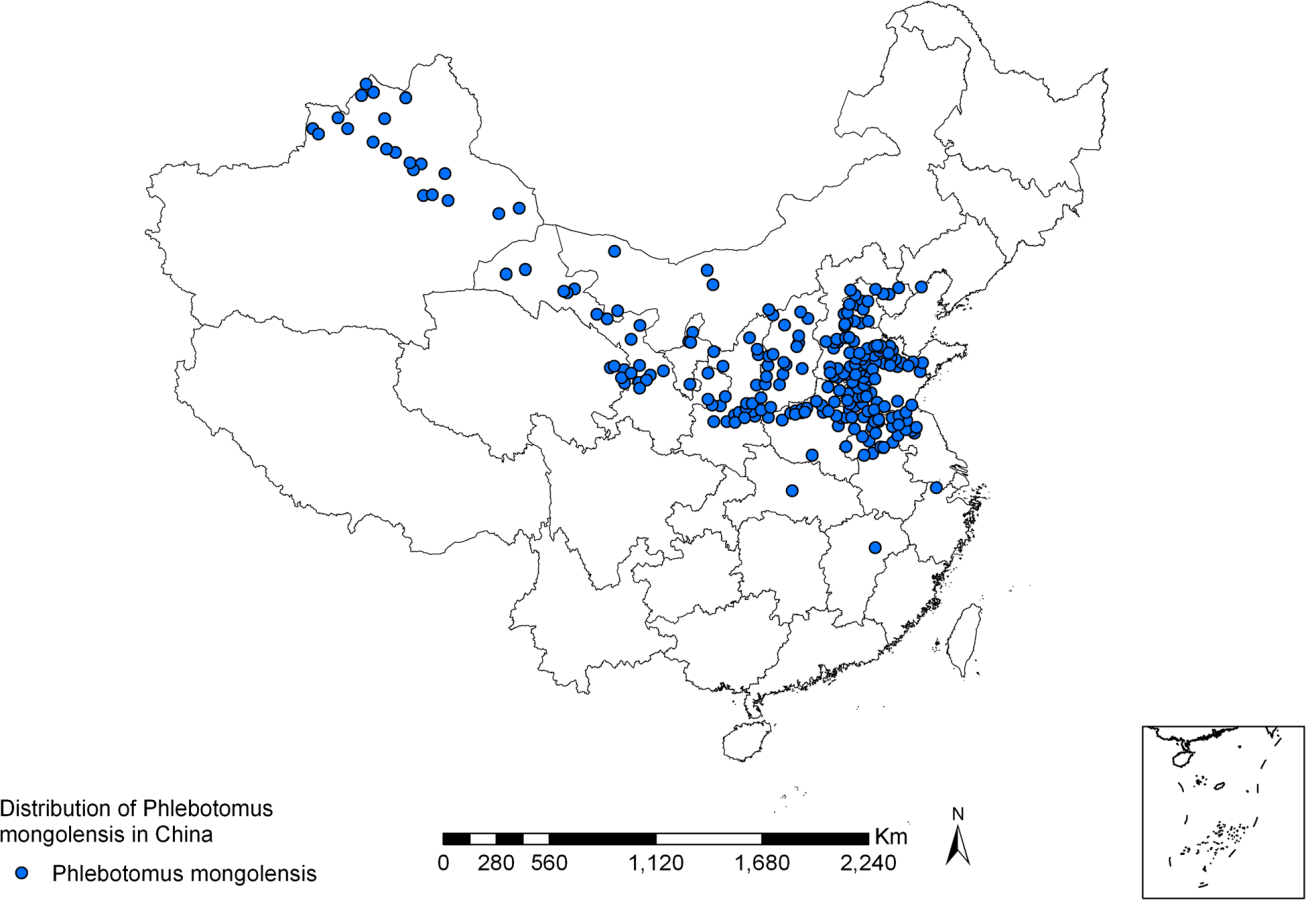
Geographic distribution of *Ph. mongolensis* sand flies in China

**Fig. 4:**
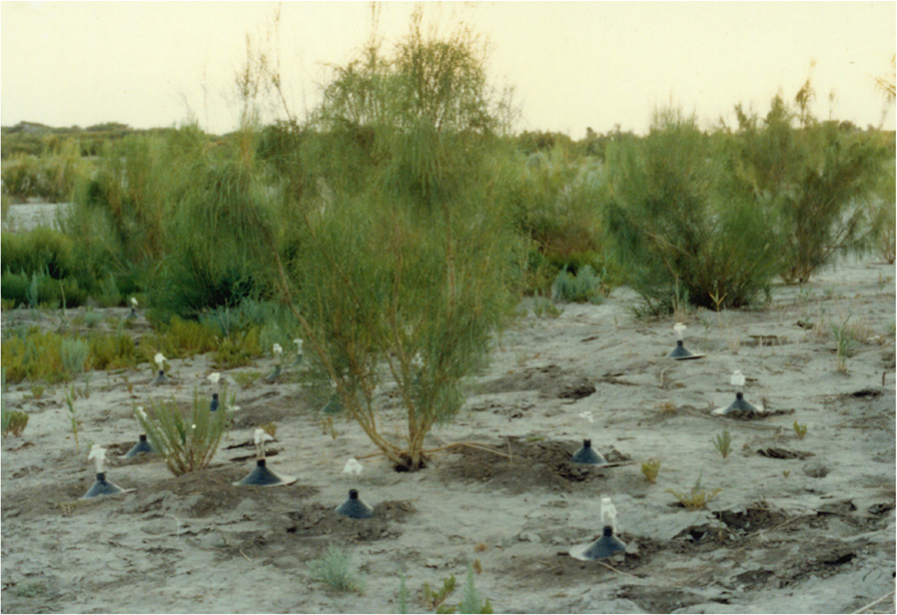
Sacsaou(*Haloxylon ammodendron*), a kind of shrub, belongs to the Chenopodiaceae family. Its developed root system is conducive to sand fixation and its leaves provide food for great gerbils (*Rhombomys opimus*). Great gerbils can climb up the branches and bite off twigs, then take the falling twigs into burrows for storage. Sacsaou-big gerbil-sand fly forms a food chain

**Fig. 5:**
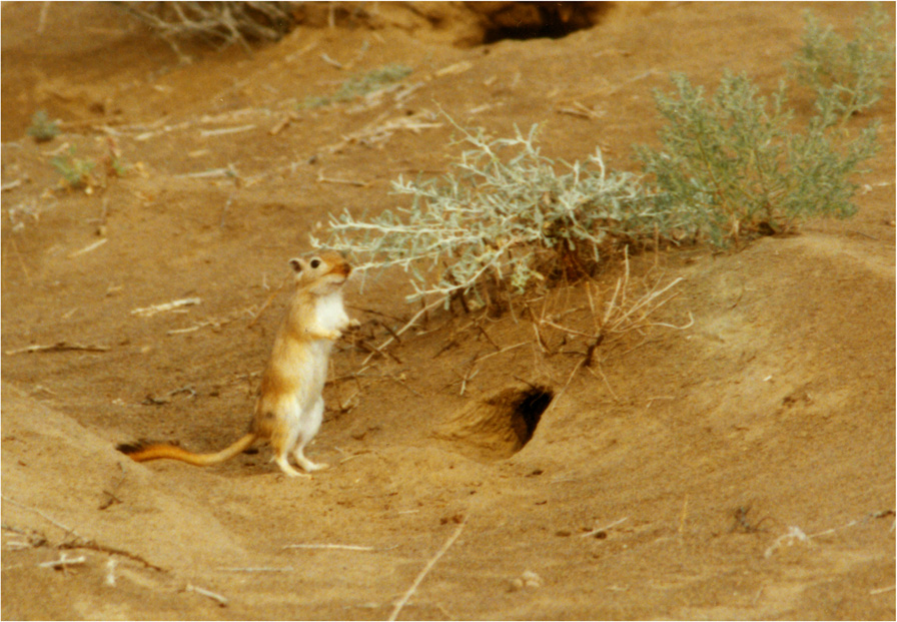
The burrows of great gerbils (*Rhombomys opimus*), the habitat of *Ph. mongolensis* sand flies, and the habitat of *Ph. andrejevi* and *Ph. caucasicus* sand flies in desert areas

*Ph. mongolensis* is distributed in 240 counties/cities in 17 provinces (autonomous regions/municipalities) [3, 4, 26, 27]. These sand flies can be found as far north as Hebukesaier, Xinjiang (46°80′N, 85°70′E), as far south as Jingmen, Hubei province (31°N, 112°10′E), as far west as Huocheng, Xinjiang (44°N, 80°80′E), and as far east as Suizhong, Liaoning province (40°30′N, 120°30′E). *Ph. mongolensis* is distributed mainly in the northern China plain region (32°–40°N, 114°–120°′E), as well as desert areas covered with Chenopodiaceae plants in Western Inner Mongolia, the Hexi Corridor of Gansu province, and the Zhungeer basin north of Mt. Tianshan, Xinjiang. However, fewer *Ph. mongolensis* sand flies are found in the Loess Plateau (34°–40°N, 102°–114°′E). *Ph. mongolensis* is present at an altitude range of 10 m (Northern Jiangsu Coastal Plain) to 1,900 m (Dongxiang, Gansu province) vertically. *Ph. mongolensis* is the dominant species in desert areas, living in burrows of great gerbils (*Rhombomys opimus*) (see Figs. 3, 4, and 5) [28].

### Vector incrimination

Besides *Ph. chinensis, Ph. mongolensis* was found to be a common anthropophilic sand fly species in Jiangsu, Shandong, and Beijing between 1925 and 1936. This sand fly species was thus examined for its role in the transmission of VL.

In 1958, *Ph. mongolensis* sand flies were experimentally infected with *Leishmania* in Lanzhou, Gansu and Huiming, Shandong. After feeding on the blood of VL patients, VL-infected hamsters, and CVL-infected dogs, the sand flies were checked for *Leishmania* promastigotes in the midgut. The results showed that the infection rate of *Leishmania* was much lower in this species than that of *Ph. chinensis*, with the infection generally limited to the midgut of the *Ph. mongolensis* sand flies. Promastigotes disappeared from the alimentary tract, as soon as the blood was digested (see Table 2).

**Table 2:** Experimental infection of *Ph. mongolensis* sand flies with *Leishmania* obtained from VL patients or CVL-infected dogs

Year	Experimental sites (county)	Source of *Leishmania* strain	No. dissected	No.infected (%)	Distribution of promastigotes in sand flies					
					Pharynx	Esophagus	Proventriculus	Midgut	Hindgut	Reference
1926	Xuzhou, Jiangsu	VL patients	14	0	0	0	0	0	0	[2]
		VL hamsters	232	7(3.0)	0	0	0	7	0	
1927	Jinan, Shandong	VL patients	202	0	0	0	0	0	0	[12]
		VL hamsters	1,170	430(36.8)	0	0	0	430	0	
1938	Huai’an, Jiangsu	VL patients	512	6 (1.17)	0	0	0	6	0	[14]
		VL hamsters	153	50(32.7)	0	0	13	49	1	
1939	Beijing	CVL dogs	449	56(12.5)	0	0	0	56	8	[15]
1958	Lanzhou, Gansu	CVL dogs	449	58(12.9)	0	0	3	53	15	^a^
1958	Huiming, Shandong	VL hamsters	125	14(11.2)	0	0	0	14	0	^a^

^a^Annual report, Institute of Parasitic Diseases, Chinese Academy of Medical Sciences, 1958, pp. 360–364

According to Feng’s observation in Beijing, after *Ph. mongolensis* fed on blood of CVL-infected dogs, promastigotes remained encased with blood in the peritrophic membrane and decreased in number as the peritrophic membrane shrank. Six to seven days later, residual blood and a small number of promastigotes in the membrane was discharged through the anus. Consequently, it can be inferred that *Ph. mongolensis* is not a permissive vector of transmitting VL in China [29].

Feng *et al.* also found the trapping of *Leishmania* in the peritrophic membrane of *Ph. mongolensis* sand flies after feeding on blood, in Lanzhou and Huiming in 1958 (Annual report, Institute of Parasitic Diseases, Chinese Academy of Medical Sciences, 1958).

On the other hand, *Ph. mongolensis* sand flies have been proven to be vectors of *Leishmania gerbilli* and *L. turanica,* which infect the subcutaneous tissue of *Rhombomys opimus* ears in the desert parts of western China [30–33]. After feeding on gerbil’s blood infected with *L. turanica,* about a quarter of *Ph. mongolensis* sand flies had their peritrophic membrane ruptured during blood meal digestion, and promastigotes continued to develop and reproduce in their midguts. Five days after feeding on blood, *L. turanica* promastigotes were found in the esophagus of the sand flies [34].

### Ph. longiductus

#### Geographic distribution

**Fig. 6:**
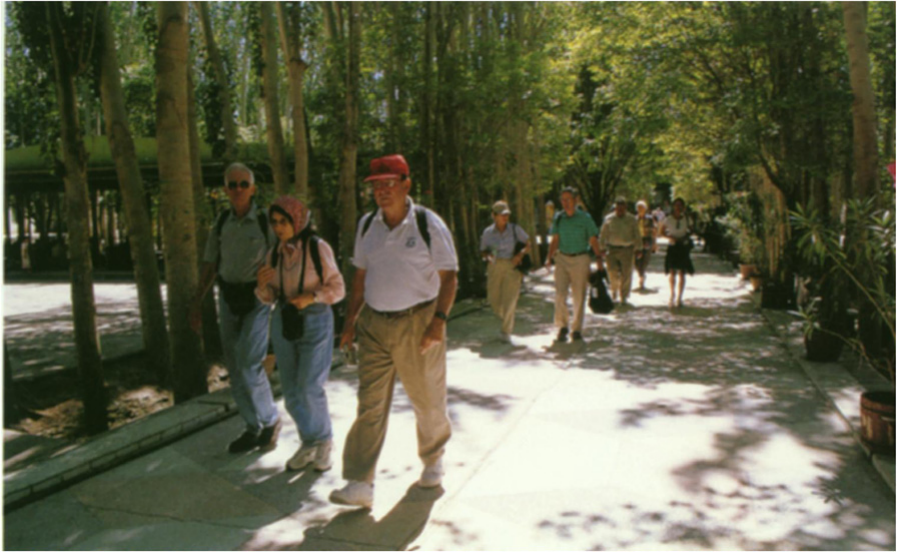
Ancient oasis, with high density population, lush vegetation, and orchards in each household, is agricultural bases in Xinjiang (photo by Xiao-kun Ding)

The distribution of *Ph. longiductus* sand flies in China is limited to 30 counties (cities) in Xinjiang, covering an area as far north as Tacheng (46°45′N, 83°E), as far south as Yecheng (37°52′N, 77°24′E), as far west as Kashgar (37°50′N, 76°E), and as far east as Shanshan (42°90′N, 90°13′E) (see Fig. 1). The species has a wide distribution range in altitude, ranging from 90 m (Turfan) to 2,100 m (Wensu). *Ph. longiductus* is especially found in ancient oases, which have a history of hundreds of years, with some being more than 2,000 years old, at elevations of 1,000 to 1,500 m (see Figs. 1 and 6) in the western and northern rims of the Tarim Basin, south of Mt. Tianshan. There are few *Ph. longiductus* sand flies in the mountainous regions, but none are found in desert areas [5, 26].

### Vector incrimination

#### *Ph. longiductus* has been the major vector of VL in endemic ancient oases, with an anthropophilic habit

*Ph. longiductus* accounts for most of the sand flies (82.3–100 %) when collected by means of tube aspirators and sticky papers at four different sites in ancient oases, followed by *Ph. Wui*, then *Ph. alexandri* and *Sergentomyia sinkiangensis* (see Table 3) [35–39].

**Table 3:** Sand fly species and composition ratios in ancient oases of southern Xinjiang

Year	Study sites (county)	No. collected	Sand fly species and composition ratio(%)			
			*Ph. longiductus*	*Ph. wui*	*Ph. alexandri*	*S. sinkiangensis*
1959–1963	Atushi	5,631	4,634(82.3)	971(17.2)	21(0.4)	5(0.1)
1988–1990	Kashgar	8,382	6,947(82.9)	1,435(17.1)	0	0
1984	Wensu	4,978	4,605(92.5)	346(7.0)	17(0.3)	10(0.5)
2005	Wushi	328	328(100)	0	0	0

*Ph. longiductus*, from the above sites, is anthropophilic, feeding on both humans and livestock. Female sand flies are present in different ovary development stages, nulliparous, or parous, when collected in households during the daytime [39–43].

#### The alimentary tract of *Ph. longiductus* sand flies is suitable for the development and reproduction of *Leishmania*

Batches of *Ph. longiductus* sand flies were dissected three to nine days after exposure to hamsters (*C. barabensis*) that were infected with *Leishmania* from patients in Xinjiang, Beijing, and Shandong. The results showed that *Leishmania* from all three regions developed and reproduced in the alimentary tract of *Ph. longiductus* sand flies. The sand fly infection rate was related to the intensity of VL infection in hamsters. In infected sand flies, promastigotes were observed to migrate from the midgut into the pharynx (see Table 4), suggesting that *Ph. longiductus* sand flies are vectors of VL in Xinjiang [38, 40].

**Table 4:** Experimental infection of *Ph. longiductus* sand flies with *Leishmania* from VL patients

Experimental sites (county)	Source of *Leishmania* strain	No. dissected	No. infected (%)	Distribution of promastigotes in sand flies						Reference
				Proboscis	Pharynx	Esophagus	Proventriculus	Midgut	Hindgut	
Atushi	Xinjiang	40	7(17.5)	0	1	2	4	6	0	[40]
Wensu	Xinjiang	45	28(62.2)	1	7	21	28	28	0	[38]
Atushi	Beijing	32	31(96.8)	0	3	25	27	30	8	[40]
Atushi	Shandong	83	46(55.4)	0	3	32	43	46	9	[40]

In Kazakhstan, which neighbors Xinjiang, Dergacheva and Strelkova (1985) reported infection in hamsters bitten by *Ph. longiductus* sand flies infected with *L. donovani* [41]. In 1990, the World Health Organization (WHO) listed *Ph. longiductus* as a proven vector of VL in Kazakhstan [42], although *Ph. longiductus* sand flies naturally infected with *Leishmania* have not yet been found, pending further investigation in Xinjiang.

### Ph. wui

#### Geographic distribution

**Fig. 7:**
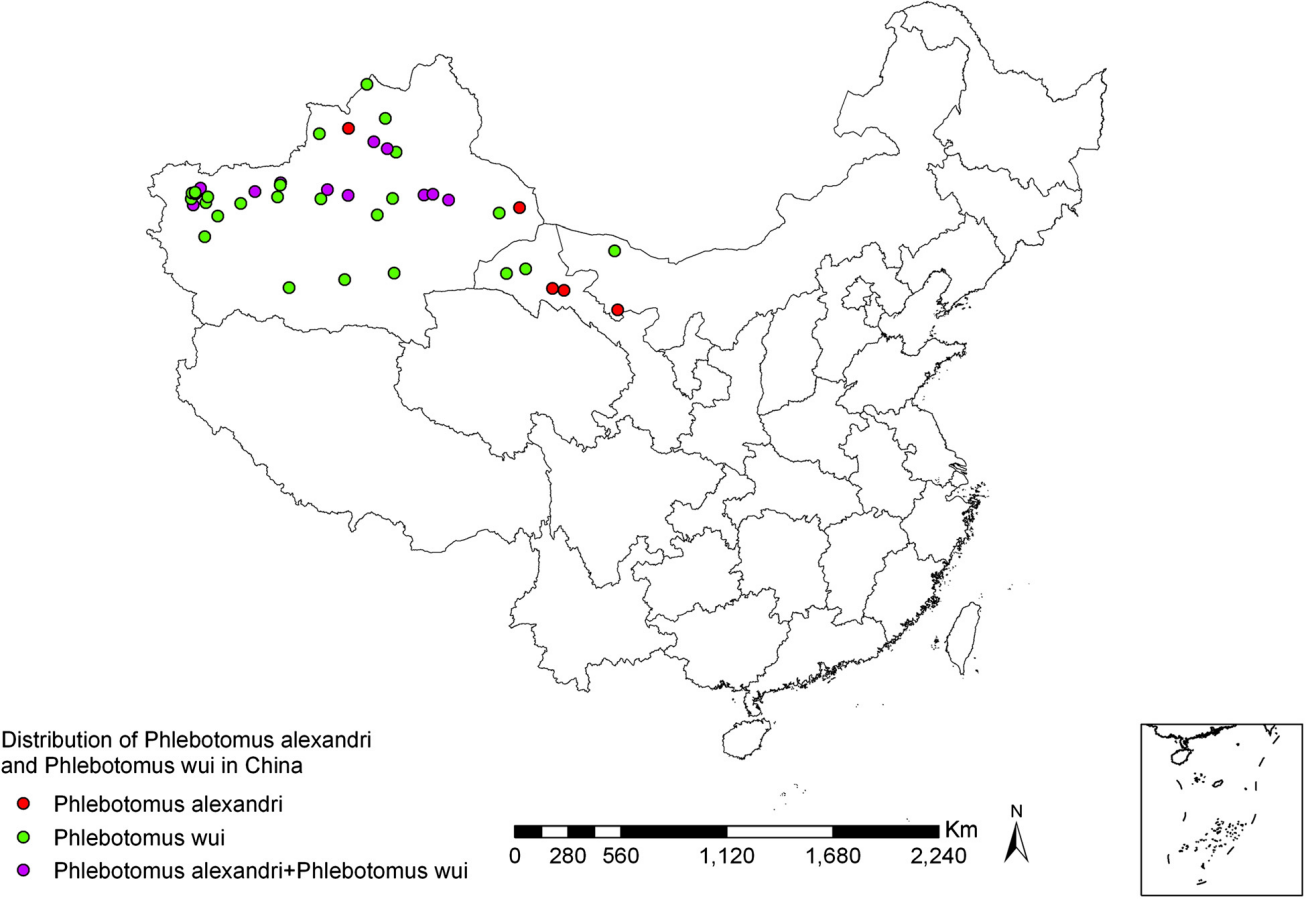
Geographic distribution of *Ph. alexandri* and *Ph. wui* sand flies in China

**Fig. 8:**
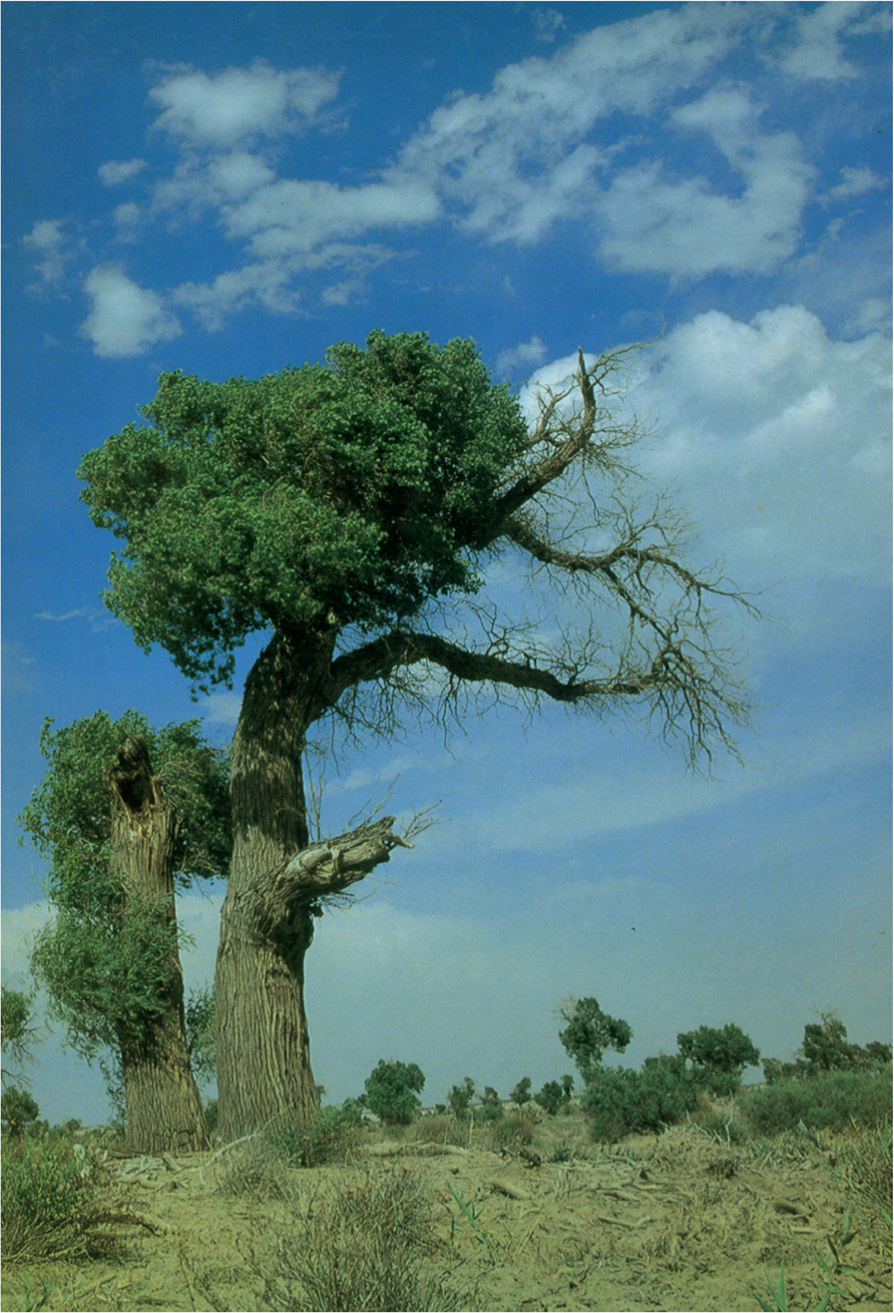
Diversiform-leaved poplar *Populus diversifolia*, old branches with cordate leaves, epicormic branches with long, narrow leaves

**Fig. 9:**
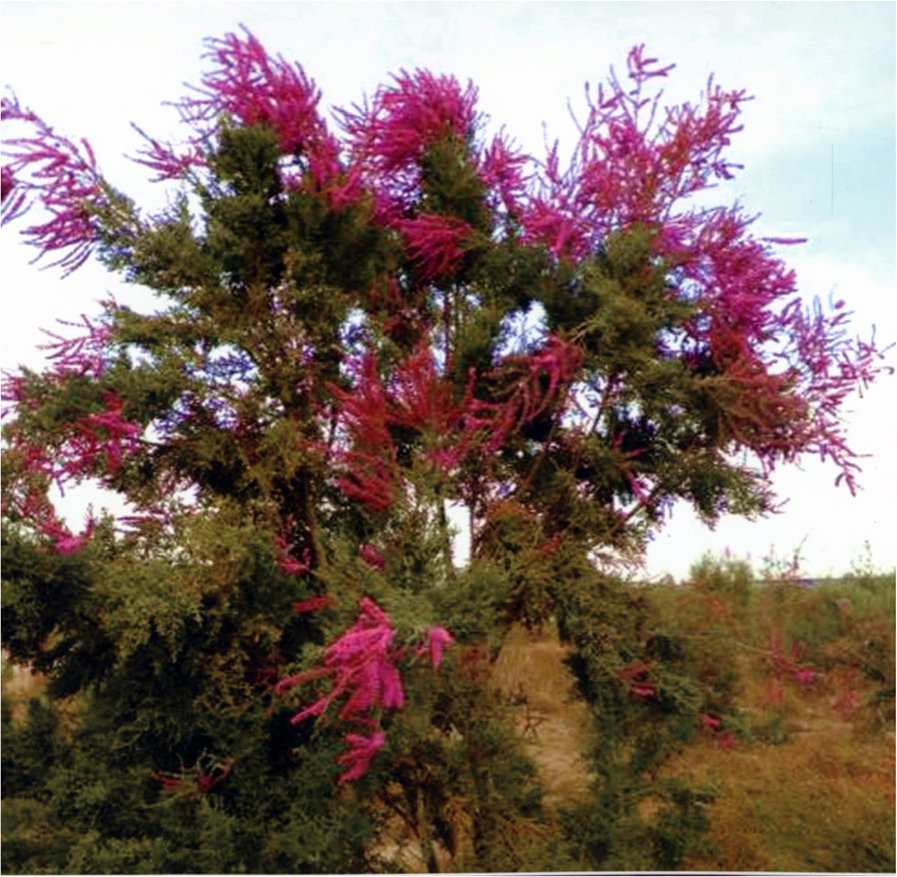
*Tamarix* is a kind of shrub with small leaves and developed roots, blooming small red flowers in the summer

*Ph. wui* sand flies are found in 37 counties (banners) in Xinjiang, Gansu, and Inner Mongolia, including in the area as far north as Tacheng, Xinjiang (46°45′N, 83°E), as far south as Minfeng, Xinjiang (37°04′N, 82°41′E), as far west as Shufu, Xinjiang (39°50′N, 75°85′E), and as far east as Eji’naqi, Inner Mongolia (41°57′N, 77°50′E) (see Fig. 7). The vertical distribution spreads from 90 m (Taoergou, Turfan, Xinjiang) to 1,500 m (Atushi, Xinjiang). *Ph. wui* is distributed at its highest density in desert areas at the edge of the Tarim Basin in south Xinjiang and western Inner Mongolia*.* The ecological habitats are mainly sites several kilometers away from rivers with lower water tables and sparse vegetation, of the *Poplar diversifolia* and *Tamarix taklamakanensis* varieties (see Figs. 8 and 9), and also in wild animal burrows, half-buried underground structures, surface collapsed pits, tree holes, and outer wall cracks of houses surrounding villages. *Ph. wui* enters rooms attracted by light at night and leaves at dawn [44–48]. When *Poplar diversifolia* dies due to river drying and desertification, *Ph. wui* sand flies become scarce or disappear completely, as is also the case in places far from rivers and without *Poplar diversifolia* vegetation [49, 50].

In general*, Ph. wui* accounts for up to 17.2 % of the total sand fly population in ancient oases, while it is rarely found in mountainous areas and not found in stony desert areas [49].

### Vector incrimination

#### Surveys have shown that *Ph. wui* was the dominant species in desert areas with vegetation of *P. diversifolia* and *T. taklamakanensis*

Surveys done in many places have indicated that only two species, namely *Ph. wui* and *S. sinkiangensis*, are present in VL-endemic deserts with *P. diversifolia* and *T. taklamakanensis* vegetation. *Ph. wui* is the dominant species, accounting for 76.4–99.9 % of total sand fly population (see Table 5).

**Table 5:** Sand fly species and composition ratios in desert areas with *P. diversifolia* and *T. taklamakanensis* vegetation, in Xinjiang and Inner Mongolia

Year	Survey sites (county)	No. dissected	Sand fly species and composition ratio(%)		Reference
			*Ph. wui*	*S. sinkiangensis*	
1963–1966	Alar, Xinjiang	29,202	22,666(77.6)	6,536(22.4)	[44]
1972	Eji’naqi, Inner Mongolia	4,492	4,429(98.6)	63(1.4)	[43]
1975–1980	Bachu, Xinjiang	683	522(76.4)	166(23.6)	[45]
2007	Minfeng, Xinjiang	1,210	1,200(99.2)	10(0.8)	[46]
2010	Jiashi, Xinjiang	4,540	4,535(99.9)	5(0.1)	[47]

*Ph. wui* sand flies feed mainly on human and other homeothermic animal blood. They frequently bite humans outdoors in the evening and also enter houses to feed on human blood. *S. sinkiangensis* is another species, which primarily feeds on lizards, with a feeding rate of 74.9 % (161/215) on lizards, and only very occasionally (1.6 %, 2/122) on humans and (0.8 %, 1/123) rats [40]. As a result, *S. sinkiangensis* is not considered to be a vector of VL [40].

#### After *Ph. wui* was artificially infected with *Leishmania* obtained from VL patients, promastigotes were observed to migrate into the pharynx

Batches of *Ph. wui* sand flies were dissected after exposure to hamsters infected with *Leishmania* obtained from VL patients in Xinjiang and being kept for three to seven days at a temperature of 22–26 °C. The promastigote infection rate was 85.1 % (211/248). In addition to the midgut, promastigotes were found in the esophagus (35.1 %, 74/211) and the pharynx (6.6 %, 14/211) of the infected sand flies on the fourth or fifth day. Moreover, they were also present in the esophagus typhlosolis and Malpighian tubules of heavily infected sand flies. The results showed that the alimentary tract of *Ph. wui* sand flies is extremely favorable for the development and reproduction of *Leishmania* obtained from VL patients [40].

#### *Leishmania* from naturally-infected *Ph. wui* sand flies is homologous to *L. infantum*

Female sand flies were collected by human-baited traps and light traps set up inside and outside of human dwellings in desert areas at night. Parous and >1/2 blood digestion sand flies were dissected and examined microscopically, giving the natural infection rate of 0.4–5.7 % [40, 43, 45, 46] (see Table 6). The distribution of promastigotes in the alimentary tract of *Ph. wui* sand flies is similar to that of experimentally infected sand flies.

**Table 6:** Promastigote natural infection in *Ph. wui* sand flies in desert areas with vegetation of *P. diversifolia* and *T. taklamakanensis* in Xinjiang and Inner Mongolia

Year	Survey sites (county)	No. dissected	No. infected (%)	Distribution of promastigotes in sand flies						
				Proboscis	Pharynx	Esophagus	Proventriculus	Midgut	Hindgut	Reference
1964	Alar, Xinjiang	300	17(5.7)	0	4	12	15	16	5	[40]
1972	Eji’naqi, Inner Mongolia	1,998	34(1.7)	0	2	16	31	34	2	[43]
1977	Bachu, Xinjiang	453	4(0.9)	2	0	3	4	4	0	[45]
2007	Minfeng, Xinjiang	523	2(0.4)	0	0	1	2	2	2	[46]

Fourteen isolates of promastigotes were taken from naturally-infected sand flies by cultivation in Novy-MacNeal-Nicolle medium. Except for the Bachu isolate [PHL(IWUI)/CN/77/771], the remaining 13 were then inoculated into the abdominal cavity and subcutaneous tissue of hamsters. Two to four months later, all infected animals suffered from serious VL, with their livers and spleens getting heavily infected [40, 43].

Genotype heterogeneity analysis of nDNA and kDNA from the Bachu isolate revealed that it is homologous to *L. infantum* [51], identical to the *Leishmania* from VL patients in similar areas [48]. *Ph. wui* is thus confirmed as a vector of VL in desert areas.

### Ph. alexandri

#### Geographic distribution

**Fig. 10:**
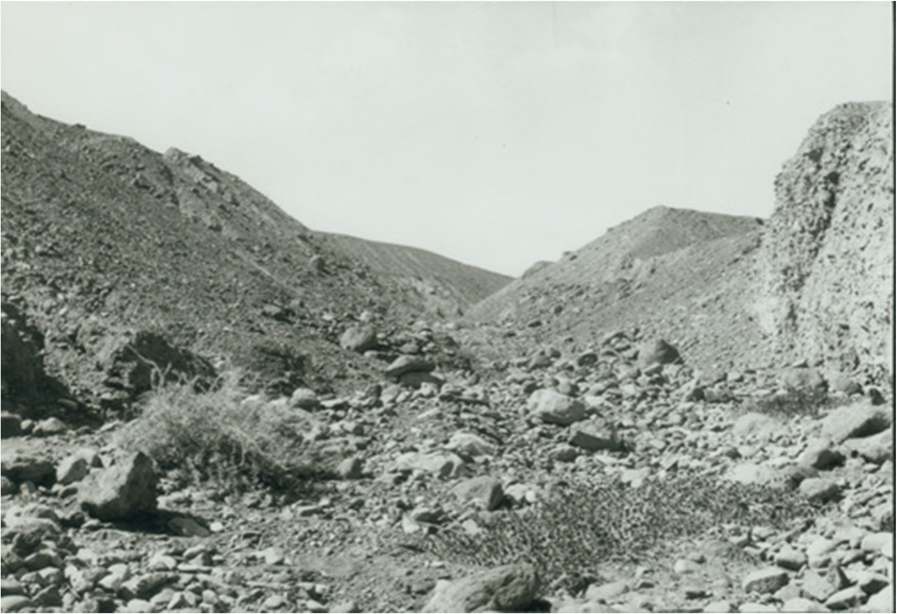
Erosion gully at the foot of stony desert terrain. In summer, the temperature can go over 50 °C during the day, and drop quickly after sunset. A large number of *Ph. alexandri* sand flies can be captured with human-baited traps in the groove

*Ph. alexandri* sand flies are distributed in 17 counties in Xinjiang, Gansu, and Inner Mongolia regions, as shown in Fig. 7. They are found in areas as far north as Guertu, Wusu, Xinjiang (44°50′N, 76°10′E), as far south as Yingjisha, Xinjiang (39°10′N, 76°10′E), as far west as Atushi, Xinjiang (39°70′N, 76°10′E), and as far east as Yabulai Mountains, Alxa Youqi, Inner Mongolia (39°40′N, 103°10′E) (see Fig. 7). *Ph. alexandri* is often present in areas of piedmont and stony desert at an altitude of 500 to 1,750 m [5]. Since stony deserts lack natural vegetation, except for small shrubs, areas get eroded due to rainstorms in summer (see Fig. 10). *Ph. alexandri* sand flies inhabit various kinds of burrows in such landscapes [52], but can also be found in dried wells at the foot of mountains [53]. *Ph. alexandri* sand flies enter residential areas at night, but rarely stays long indoors during the daytime. They frequently dwell in large caves bite to humans who enter the caves in the daytime [52]. This species is rarely found in mountainous areas and is occasionally found in oases [49].

### Vector incrimination

#### *Ph. alexandri* is the principal species in VL-endemic areas at the junction of the foot of Mt. Tianshan and the stony desert in Xinjiang

In 1983, a total of 8,843 sand flies were captured in Meiyaogou Turfan, among which *Ph. alexandri* was the dominant species, accounting for 81.1 % (7,176/8,843) of the sand flies [54]. The following year, similar results were obtained in a similar investigation in Damazha, Wensu, Xinjiang. *Ph. alexandri* sand flies constitute 91.5 % (1,704/1,863) of the total sand fly population that is caught and examined [38].

#### *Ph. alexandri* is markedly anthropophilic

Sand flies were collected for a study in Meiyaogou, providing evidence of the anthropophilicity of *Ph. alexandri*. Using a human as bait, 835 of the 837 (99.8 %) sand flies caught indoors and in caves belonged to the *Ph. alexandri* species. Another survey also indoors and in caves, all of the 213 sand flies caught that were sucking blood were identified as *Ph. alexandri* [54]*.* In the Damazha in Wensu counties, 15 of the 1,704 (0.9 %) *Ph. alexandri* sand flies were found in caves, with the remaining 1,689 found in stony desert areas at night. In the Meiyaogou, when 96 *Ph. alexandri* sand flies were kept in the same cage with two anesthetized lizards overnight, it was found that they refused the lizards’ blood [54].

#### *Ph. alexandri* sand flies are susceptible to *Leishmania* infection obtained from VL patients

*Ph. alexandri* were dissected four to twelve days after exposure to hamsters that were infected with *Leishmania* obtained from VL patients in Xinjiang. Promastigotes invaded the esophagus and pharynx on days four to six, and the proboscis on the ninth day. Promastigotes formed rosettes in the junction of the hindgut and Malpighian tubes in some of the heavily infected sand flies. This showed that the *Leishmania* infection is extremely adaptable to the alimentary tract of this species (see Table 7). Furthermore, it was reported that *Ph. alexandri* sand flies were also susceptible to *Leishmania* from VL patients in Henan and Gansu provinces. After feeding on hamsters infected with *Leishmania*, promastigotes flourished in the midgut of this species, and invaded the pharynx on the eighth day [55].

**Table 7:** Experimental infection of *Ph. alexandri* sand flies with *Leishmania* obtained from VL patients

Source of sand flies (county)	No. dissected	No. infected (%)	Distribution of promastigotes in sand flies							
			Proboscis	Buccal cavity	Pharynx	Esophagus	Proventriculus	Midgut	Hindgut	Reference
Meiyaogou	245	230(93.9)	12	3	67	175	229	230	13	[54]
Damazha	47	40(85.1)	1	1	8	34	40	40	2	[38]

#### Natural infection of *Ph. alexandri* with promastigotes

A total of 643 and 386 *Ph. alexandri* sand flies were collected outdoors and indoors in Meiyaogou and Damazha, respectively. They were dissected after complete blood digestion for microscopic examination. Thirteen (2.02 %) and four (1.04 %) sand flies from Meiyaogou and Damazha, respectively, were found to be infected with promastigotes. Six heavily infected sand flies showed promastigotes congested in the midgut, the pharynx, the buccal cavity, and the proboscis. The results were consistent with that of an artificial infection [38, 54]. Promastigotes from seven sand flies were isolated and inoculated into the peritoneal cavity and skin of seven normal hamsters. The hamsters were examined 88–97 days later, with infection by amastigotes observed in all of them [54].

Promastigotes isolated from the sand fly midgut proliferate in the Novy-MacNeal-Nicolle medium. One isolate (IALE/CN/88/Turfan10) was identified as *L. donovani* (zymodeme: MON-138) using isozyme electrophoresis detection (J.A. Rioux, 1991, unpublished data) by *Laboratoire d’Ecologie médicale et de Pathologie parasitaire* in Montpellier, France. At the same location, another isolate (IALE/CN/87/self-1) was identified as *L. infantum* through nDNA and kDNA genotype analysis [51], which was highly homologous to the WHO reference strain *L. donovani* (MHOM/IN/80/DD8) through repetitive DNA sequence homology analysis [56]. The difference may be attributed to the different methods used.

#### Experimental transmission of *Leishmania* to normal hamsters via the bite of *Ph. alexandri* sand flies, infected with *Leishmania* obtained from VL patients

Two batches of sand flies (one with 18 and the other with 23 sand flies) fed on a hamster that had been infected with *Leishmania* from VL patients. Eleven to twelve days later, two normal hamsters were exposed to these sand flies. Only three sand flies fed on the blood from hamster no. 1 and seven fed on hamster no. 2. These 10 fed sand flies were dissected, with promastigotes found in six of them. Among them, five sand flies were heavily infected with promastigotes in the proventriculus, and even in the pharynx and proboscis. Hamster no. 1 was dissected on the 147th day and hamster no. 2 hamster on the 145th day. Amastigotes were found in the smears of the liver, spleen, and lymphonodus of both hamsters, demonstrating that both developed VL [54]. These results suggest the capacity of *Ph. alexandri* sand flies as vectors of VL.

### Conclusions

Past investigations have shown that vectors of VL vary in different geographic landscapes in VL-endemic areas of China. *Ph. chinensis* sand flies are the dominant and the most important vectors in mid-east plain, mountainous, and Loess Plateau areas in China. In the vast region stretching from western Inner Mongolia to Xinjiang, *Ph. longiductus* sand flies prevalent in ancient oases are a major vector of VL. *Ph. wui* and *Ph. alexandri* sand flies are vectors of VL in deserts with vegetation of *P. diversifolia* and *T. taklamakanensis*, and at the foot of mountains in stony deserts, respectively.

*Ph. mongolensis* sand flies are widely distributed in central and eastern plains of the country. *Leishmania* isolated from VL patients replicates, but fails to break free from encapsulation of the peritrophic membrane, when human blood is digested completely in the midgut of this species. *Ph. mongolensis* sand flies are thus not vectors of human leishmaniasis. However, the species are a proven vector transmitting *L. gerbilli* and *L. turanica,* which parasitize subcutaneous tissues to produce ear lesions in the great gerbil (*Rhombomys opimus*) [30, 31, 33]. It has been elucidated which sand fly species are vectors of VL in major epidemic areas in China, but this is less definitive in areas with sporadic VL cases, such as the ones in deep valleys in Mt. Tianshan, Xinjiang, and areas in Dunhuang and Guazhou, Gansu. These areas need to be investigated further.

The four sand fly species that are vectors of VL in China are distributed in different geographic areas that have different ecological features. Further studies that elucidate the relationship between the geographic distribution pattern of sand flies and their natural environment—climate, physical and chemical properties of soil, vegetation, ground temperature, and annual precipitation—are needed in order to foster VL control and surveillance.

In addition, cutaneous leishmaniasis (CL) has often been diagnosed among workers who returned from the Middle East and North Africa [57, 58]. Cases of VL and CL have also been reported among visitors from Western Europe and South America [59, 60]. Research should be conducted on whether *Leishmania* brought by these imported cases to China can be transmitted by indigenous sand fly vectors. In recent years, because the electric light has become more widespread, a large number of *Ph. wui* sand flies with strong phototaxis enter dwellings at night, attracted by light, from the surrounding deserts in ancient Kashgar oasis, south of Xinjiang [61]. Further studies on the role of *Ph. wui* sand flies in the transmission of VL in these regions are thus needed.

## Additional file

Additional file 1:
**Multilingual abstracts in the six official working languages of the United Nation.** (PDF 384 kb)

## Abbreviations

CL: Cutaneous leishmaniasis
CVL: Canine visceral leishmaniasis
VL: Visceral leishmaniasis
WHO: World health organization
