# Supplementary material for: Phlebotomine sand flies (Diptera: Psychodidae) transmitting visceral leishmaniasis and their geographical distribution in China: a review
Source: Infect Dis Poverty. 2016 Feb 23;5:15. doi: 10.1186/s40249-016-0107-z (PMC4765030; doi:10.1186/s40249-016-0107-z)
Supplement: Additional file 1: — Multilingual abstracts in the six official working languages of the United Nation. (PDF 384 kb) [file 40249_2016_107_MOESM1_ESM.pdf]

Translation of the abstract into the six official working languages of the United Nations

ذبابة الرمل (ذوات الجناحين: فراشيات المظهر) الناقلة لداء الليشمانيات الحشوي وتوزيعها الجغرافي في الصين: مراجعة

لى رن جوان، تشنج تشو-بن، تشانج فا جين، تشينج فو، جون جي تشاي

ملخص

منذ ورود أنباء عن وجود ذبابة الرمل لأول مرة في الصين في عام 1910، دُرس توزيع الأنواع المختلفة ودورها في نقل داء الليشمانيات الحشوي (VL)على نطاق واسع. وحتى عام 2008، تم التحقق من أن أربعة أنواع هي الحاملة للـVL، وهي Phlebotomus chinensis (Ph. sichuanensis) وPh. longiductus (Ph. chinensis longiductus) وPh. wui (Ph. major wui) و Ph. Alexandri.
تختلف أنواع ذبابة الرمل كثيرا وفقا للبيئات الطبيعية في المناطق الجغرافية المختلفة حيث أنها متوطنة. تعيش Ph. Chinensis في نطاق واسع وقابلة للتكيف مع بيئات مختلفة، وبالتالي تنتشر على نطاق واسع في المناطق المدنية، والجبلية، وهضبة اللوس شمال نهر يانجتسى. تنتشر Ph. longiductus بشكل رئيسي في مناطق الواحات القديمة جنوب جبل تيانشان في منطقة شينجيانج الويغورية ذاتية الحكم. Ph. wui هي النوع السائد في الصحراء ذات الغطاء النباتي من شجر الحور والأثل في شينجيانج والجزء الغربي من منطقة منغوليا الداخلية. وأخيرا، Ph. alexandri صعبة التكيف تتواجد فقط في المناطق الصحراوية الصخرية، مثل سفوح الجبال في شينجيانج وغرب ممر خشي، في مقاطعة جانسو. تلخص هذه المراجعة العلاقة بين نمط التوزيع الجغرافي لأنواع ذبابة الرمل الأربعة وشكل بيئتها من أجل تعزيز البحوث حول توزيع المرض وتخطيط مكافحة ذبابة الرمل. وعلاوة على ذلك، تمت مناقشة بعض المشاكل التي لا يزال يتعين حلها عن ناقلات داء الليشمانيات الحشوي في الصين .

Translated from English version into Arabic by Mahmoud Sami, through

TRANSLATORS WITHOUT BORDERS

中国传播内脏利什曼病的白蛉（双翅目:毛蛉科）及其地域分布的调查研究

Li-Ren Guan, Zheng-Bin Zhou, Chang-Fa Jin, Qing Fu, Jun-Jie Chai

摘要

本文介绍了中国境内内脏利什曼病媒介的研究过程和获得的结果。迄今已有四种白蛉---中华白蛉（*Phlebotomus chinensis*）、长管白蛉（*Ph. longiductus*）、吴氏白蛉（*Ph. wui*）和亚历山大白蛉（*Ph. alexandri*）已被证实为内脏利什曼病的传播媒介。

对多年来的调查研究资料分析表明，各地自然环境不同，白蛉的种类组成亦有所差异，不同蛉种有其不同的地域分布格局。中华白蛉对环境条件有广泛适应能力，在长江以北的平原、山丘和黄土高原地带均有其分布；长管白蛉的主要分布区是新疆天山南部的古老绿洲地带；吴氏白蛉是新疆和内蒙古西部胡杨—柽柳荒漠地带的优势种；亚历山大白蛉则主要分布在新疆以及甘肃河西走廊西部的山麓与砾质荒漠相衔接的地带。不同的地理景观地带有不同的蛉种，内脏利什曼病媒介也因地而异。本文归纳了该四种媒介白蛉的地域分布格局与地理景观之间的关系，进而为内脏利什曼病的地理分布模式的研究和拟定白蛉防制规划提供参考。并对有关中国内脏利什曼病媒介尚待研究的一些问题也做了讨论。

Translated from English version into Chinese by Zhou Zhengbin

Les phlébotomes (Diptera : Psychodidae) transmettant la leishmaniose viscérale et leur distribution géographique en Chine : revue

Li-Ren Guan, Zheng-Bin Zhou, Chang-Fa Jin, Qing Fu, Jun-Jie Chai

Résumé

Depuis que leur présence en Chine a été rapportée pour la première fois en 1910, la distribution des différentes espèces de Phlebotominae et leur rôle dans la transmission de la leishmaniose viscérale ont été abondamment étudiés. À la date de 2008, le rôle de quatre espèces comme vecteurs de la leishmaniose viscérale a été confirmé : *Phlebotomus chinensis* (*Ph. sichuanensis*), *Ph. longiductus* (*Ph. chinensis longiductus*), *Ph. wui* (*Ph. major wui*) et *Ph. alexandri.*

Les espèces de phlébotomes varient beaucoup selon l'environnement naturel dans les différentes régions géographiques où elles sont endémiques. *Ph. chinensis* est euryèce et s'adapte à différentes écologies . on le trouve ainsi largement distribué dans les plaines, les montagnes et les plateaux de lœss au nord du fleuve Yangzi. *Ph. longiductus* se trouve principalement dans les anciennes régions d'oasis au sud des monts Tianshan, dans la Région autonome Ouïgoure du Xinjiang. *Ph. wui* est l'espèce prédominante dans les déserts à *Populus diversifolia* et *Tamarix* du Xinjiang et dans la partie occidentale de la Région autonome de Mongolie Intérieure. Enfin, *Ph. alexandri* est sténoèce et ne se trouve que dans les zones de déserts pierreux, par exemple au pied des montagnes du Xinjiang et dans l'ouest du Corridor du Hexi, dans la province du Gansu. Notre revue a résumé les liens entre le schéma de distribution géographique des quatre espèces de phlébotomes et leur biotope géographique, afin de faire avancer la recherche sur la distribution de la leishmaniose viscérale et la planification de la lutte contre les phlébotomes. Elle aborde également quelques problèmes qui restaient à résoudre à propos des vecteurs de leishmaniose viscérale en Chine.

Translated from English version into French by Suzanne Assenat, through

TRANSLATORS
WITHOUT BORDERS

Переносчики висцерального лейшманиоза москиты-флеботомусы (Diptera: Psychodidae) и их географическое распространение в Китае: обзор

Ли-Жень Гуань, Чжэн-Бинь Чжо, Чань-Фа Цзинь, Цинь Фу, Цзюнь-Цзе Чай

Аннотация

Распространение различных видов москитов-флеботомусов и их роль в передаче висцерального лейшманиоза (VL) широко изучались после первого сообщения о них в Китае в 1910 г. Вплоть до 2008 г., в качестве переносчиков VL были подтверждены четыре вида, а именно: *Phlebotomus chinensis* (*Ph. sichuanensis*), *Ph. longiductus* (*Ph. chinensis longiductus*), *Ph. wui* (*Ph. major wui*) и *Ph. alexandri.*

Виды москитов широко меняются в зависимости от природной среды в различных географических районах, где они являются эндемичными. *Ph. Chinensis* является эврибионтом, адаптируется к различным условиям окружающей среды и поэтому широко распространен на равнине, в горах и регионах Лессового

плато севернее реки Янцзы. *Ph. Longiductus*, в основном, распространен в зонах древних оазисов южнее гор Тянь-Шань в Синьцзянь-Уйгурском автономном районе. *Ph. Wui* является преобладающим видом в пустынях с растительностью *Populus diversifolia* (*вид тополя*) и *Tamarix (тамариск, гребенщик)* западной части автономного района Внутренняя Монголия. И, наконец, *Ph. Alexandri* является стеробионтом и обнаруживается только в каменистых пустынных районах, таких, как подножия гор в Синьцзяне и западный Коридор Хэси в провинции Ганьсу. В данном исследовании резюмирована зависимость между паттерном географической распространенности четырех видов москитов и их географического ландшафта с целью способствования исследованию распространения заболевания и планированию борьбы с москитами. Кроме этого, рассмотрены некоторые проблемы, связанные с переносчиками VL в Китае, которые еще предстоит решить.

Translated from English version into Russian by Alexander Somin, through

Las mosca de la arena o flebotomos *(díptera: Psychodidae*) y la transmisión de la leishmaniais visceral según su distribución geográfica en China

Li-Ren Guan, Zheng-Bin Zhou, Chang-Fa Jin, Qing Fu, Jun-Jie Chai

Resumen

Desde 1910, año en que se reportó por primera vez la existencia de moscas de la arena de la subfamilia *phlebotominae* en China, la distribución de las diferentes especies y su papel en la transmisión de la leishmaniasis visceral (VL) ha sido objeto de extensos estudios. Desde entonces y hasta 2008, se han identificado estas cuatro especies como vectores comprobados de la enfermedad: *Phlebotomus chinensis* (*Phlebotomus.sichuanensis*), *Phlebotomus.longiductus* (*Phlebotomus.chinensis longiductus*), *Phlebotomus wui* (*Phlebotomus major wui*) y *Phlebotomus alexandri.*

Las especies de mosca de la arena varían en gran medida en función del entorno natural de las diferentes zonas geográficas de donde son endémicas. La *Phlebotomus chinensis* es euritópica y se adapta fácilmente a distintos hábitats, por lo que se distribuye ampliamente por las zonas llanas y de montaña al norte del río Yangtsé y en la meseta de Loess. La *Phlebotomus longiductus* se encuentra distribuida principalmente en antiguas zonas de oasis al sur de las montañas de Tian Shan, en la Región Autónoma Uigur de Xinjiang. La *Phlebotomus wui* es la especie predominante en las zonas de desierto con vegetación *Populus diversifolia* y *Tamarix* de Xinjiang y del oeste de la Región Autónoma de Mongolia Interior. Por último, la *Phlebotomus alexandri* es estenotópica y se halla solo en zonas desérticas pedregosas, como al pie de las montañas de Xinjiang y en la parte occidental del Corredor de Hexi, en la provincia de Gansu. Esta revisión resume la relación entre el modelo de distribución geográfica de estas cuatro especies de flebótomos y su entor6no natural, con el objetivo de promover la investigación de la distribución de la enfermedad y la planificación del control de la especie transmisora. También se tratan algunos de los problemas pendientes de resolución relacionados con los vectores de la leishmaniasis visceral en China.

Translated from English version into Spanish by Marta Callava Linares, through

TRANSLATORS
WITHOUT BORDERS
